# Mathematical validation of measurement of root fracture resistance: an in vitro study

**DOI:** 10.1186/s12903-021-01873-w

**Published:** 2021-10-07

**Authors:** Rafat Bagheri, Abbas Abbaszadegan, Mohammad R. Nabavizadeh, Maryam Ferooz, Peter Parashos

**Affiliations:** 1 Dental Materials Department and Biomaterials Research Center, School of Dentistry, Shiraz University of Medical Sciences, Shiraz, Iran; 2 Department of Endodontics, School of Dentistry, Shiraz University of Medical Sciences, Shiraz, Iran; 3 Royal Australian Air Force, RAAF Base Williamtown, Williamtown, NSW Australia; 4 Melbourne Dental School, The University of Melbourne, 720 Swanston St, Melbourne, VIC 3010 Australia

**Keywords:** Fracture load, Surface area, Root canal volume, Root volume, Rotary NiTi

## Abstract

**Background:**

The aim of this study was to develop a mathematically valid method of assessing fracture resistance of roots. The model developed used mesial roots of lower molars instrumented using stainless steel hand files (SS) and two rotary nickel-titanium (NiTi) systems.

**Methods:**

Eighty human lower molars were selected and randomly divided into four groups (n = 20). After instrumentation, the root canals were obturated using thermoplasticized gutta percha. The roots were covered with a simulated periodontal ligament and mounted vertically in autopolymerizing acrylic in PVC tubes. Using a universal testing machine, the force to fracture (N) was applied and the maximum load (FL) was recorded. Remaining dentine volume was calculated and the fracture resistance (FR) was recorded. The data were analyzed using SPSS version 22 with P < .05.

**Results:**

There were no significant differences among the instrumentation methods for FL but in FR the roots instrumented using rotary NiTi showed significantly lower values than control groups and SS files (P < 0.001).

**Conclusions:**

Considering the effect of root length, volume of the root, and volume of the instrumented canal as well as the maximum failure load may be a more objective method of reporting fracture resistance of roots.

## Background

Compared with vital teeth, root canal treated roots have a higher risk of fracture [1]. Endodontic treatment procedures such as access cavity, root canal preparation, irrigation and obturation, may result in weakened dentinal walls and predispose roots to vertical fracture [2, 3]. Applying mechanical forces during root canal instrumentation may increase strain and possibly create microcracks at the root surface [4, 5]. Reasons for the weakening of roots during root canal preparation using NiTi instruments include greater tapers [1, 6, 7], instrument size [4], rotational forces applied to the root canal walls [8], preparation motion and the cross-sectional design of the instruments [9]. However, some authors [10] found no significant difference in the fracture load of hand and rotary NiTi canal preparations possibly due to the wide range of randomly collected extracted teeth that had different root canal morphology, age and restorative history.

Despite several studies reporting the effect of NiTi instrumentation on the fracture resistance of teeth [9–11], only one [11] considered the effect of root dimensions. The majority of the aforementioned studies have only considered the maximum load to fracture as the fracture resistance, which is technically inaccurate because fracture resistance is defined as “the maximum load over the surface area of the root” [12]. Therefore, the aim of this study was to develop a more objective method for reporting fracture resistance of roots, using a novel model to evaluate the fracture resistance of mesial roots of lower molars instrumented with two rotary NiTi systems and stainless steel hand instruments.

## Methods

Eighty mature human mandibular first and second molars with mesial root curvature within the range of 20°–40° were selected from an existing pool of extracted human teeth in the Biomaterial Research Center, Shiraz, Iran. Root curvatures were measured after pre-operative radiographs according to the methodology of Schneider [13]. Teeth were excluded if they had heavily calcified canals, canals with apical foramina larger than a size 15 hand file (i.e. less than a size 20 file) as assessed externally, or with preexisting fractures or cracks when examined under a light microscope at × 20 magnification (Dino-Lite Pro2 AD413TL; AnMo Electronics Corp, New Taipei City, Taiwan). The teeth were stored in 0.1% chloramine T solution at 4 °C throughout the study.

After access cavity preparation, a size 10 K-file (MANI, INC. Utsanomiya, Japan) was placed into the canal until it was visible at the apical foramen and 1 mm was subtracted to establish working length (WL). A glide path was then prepared to a size 15 K-file. Each canal was irrigated with 2 mL of a freshly prepared 1% NaOCl solution between each instrument and dried with paper points. The teeth were then randomly divided into the following four groups (n = 20).

### Group 1—Non-instrumented root canals (control group)

Root canals were only irrigated as far into the canal as feasible; there was no glide path negotiation, instrumentation, or obturation.

### Group 2—Instrumentation with stainless steel hand files (SS)

Canal preparation was performed using a step-back technique with the apical portion enlarged up to a size 25 K-file (MANI, INC. Utsanimiya, Japan). Progressively larger K-files were used to step-back in 1 mm increments to 5 mm short of WL. Gates Glidden (GG) drills (MANI, INC. Utsanimiya, Japan) were used to enlarge the middle and coronal portions of the root canals as follows: GG #2 to 5 mm short of the WL, GG #3 to 7 mm short, and GG #4 was used just into the orifices.

### Group 3—Instrumentation with ProTaper Next (PTN)

Canals were prepared using PTN rotary NiTi instruments (Dentsply Maillefer, Ballaigues, Switzerland) using an adjustable torque- and speed-controlled endodontic motor (Endo-Mate DT; NSK Nakanishi, Inc, Kanuma, Japan) according to the manufacturer’s instructions. In brief, X1 (0.17/0.04) and X2 (0.25/0.06) instruments were sequentially used to WL in a crown-down manner.

### Group 4—Instrumentation using Mtwo (M2)

Canals were instrumented using a modified Mtwo (VDW GmbH, Munich, Germany) protocol to a standardised 35/0.04 instrument, after initial crown-down preparation with the 25/0.07 (5 mm short of WL) and 30/0.05 (2 mm short of WL) instruments. The same electric motor was used as for the PTN, and torque settings were selected for each instrument according to the manufacturer’s instructions. In all groups, each instrument was used only once.

### Obturation

After SS preparation, root canals were dried and obturated with minimal force using thermoplastic compaction (BeeFill® 2in1, VDW GmbH, Munich, Germany). After the preparation was completed for the NiTi groups, the roots were filled with their respective gutta-percha (GP) systems (GP size matched with the master apical file) using a single-cone technique and 2Seal easymiX® (VDW GmbH, Munich, Germany) as a canal sealer.

Postoperative radiographs were taken in bucco-lingual and mesio-distal directions to confirm the adequacy of the root canal obturation. The obturated teeth were examined again using the microscope to exclude any teeth with cracks that may have been created during the treatment.

### Mounting the roots and measuring fracture resistance

The mesial roots were sectioned at orifice level using diamond discs under water-cooling leaving a length of approximately 12 mm. To calculate true fracture resistance (FR), i.e. Force/Surface area [12], root length and volume of the roots were considered according to the equation: SA = 3(V1-V2)/h where SA = surface area, h = root length, V1 = volume of the root and V2 = volume of the canal.

**Fig. 1:**
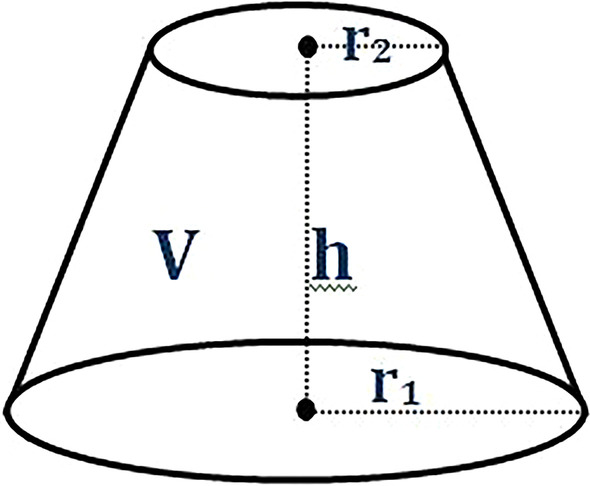
Volume (V) of a truncated cone, where h is the height, r_1_ the maximum radius and r_2_ the minimum radius

The root volume was measured by taking a silicone impression of the outer surface of the root up to the CEJ. This was then weighed on an electronic balance (GR-3000, A & D CL Toshiba, Tokyo, Japan) to an accuracy of 0.1 mg and filled with water. The amount of water required to fill the silicone mould was also weighed and subtracted from the volume of the mould to determine the total volume of the root (V1). Because the shape of the root canal preparation was essentially a truncated cone (Fig. 1), the volume of the root canal space (V2) was based on the canal taper and apical size for each instrumentation method and calculated using the following equation: V2 = πh(r_1_^2^ + r_1_r_2_ + r_2_^2^) / 3, where h is the height (i.e. length of root), r_2_ is half the apical preparation size, and r_1_ is the radius at that plane of the root based on the dimensions of the final instrumentation system.

All the roots were mounted vertically in autopolymerizing acrylic in PVC tubes with diameter 20 mm and length 20 mm. The roots were covered with a 1 mm layer of light body polyvinyl siloxane (Affinis, Coltene AG/Whaledent Inc, Altstatten, Switzerland) to simulate a periodontal ligament and positioned in the center of the acrylic resin with 3 mm of the coronal root portion exposed. The mounted specimen was placed in a jig and aligned vertically in the universal testing machine (Zwick/ Roell Z020; Zwick GmbH & Co, Germany). A vertical loading force to fracture was applied using a cone-shaped metal rod (0.7 mm diameter blunt tip) mounted on the machine directly over the canal opening of each root at a rate of 1.0 mm per min. The force, measured in Newton (N), was recorded and the maximum load was designated as the fracture load (FL).

### Statistical analysis

The collected data were analysed using the SPSS package (version 22, SPSS Inc., Chicago, IL, USA). The normality assumption was assessed using the Kolomogorov–Smirnov test. The assumption of homogeneity of variances for data in the FR group was not supported; therefore a natural logarithm was applied to make the variation of data homogenous. A one-way ANOVA with post-hoc Duncan’s test was used for each value group (FL and FR) to compare the three instrumentation methods and the control group, with *P* < 0.05.

## Results

The root canal preparations, from which the volumes (V2) were calculated, resulted in the following apical sizes and tapers—25/0.05 for SS hand files, 35/0.04 for the modified M2 protocol, 25/0.06 for PTN and 20/0.02 in the control group. The dimensions of the control group root canals were based on an assumption following the selection of canals with apical sizes less than a size 20 K-file.

The mean values and standard deviations obtained from two different calculations (FL and FR) for all preparation methods are presented in Table 1. There were no statistically significant differences in FL among the instrumentation methods. The FR of the roots in the control group was significantly higher than all other groups (*P* < 0.001). The second highest value was related to the roots instrumented using M2, followed by SS hand files and PTN, but with no statistically significant differences.

**Table 1:** Mean values ± standard deviation (SD) of fracture loads of the roots (FL), fracture resistance (FR) and natural logarithm of FR (Ln/FR)

Instrumentation methods	n	FL (N)	FR, (MPa)	Ln/FR
Control	20	410.16 ± 80^A^	2177.47 ± 348^A^	8.15 ± 0.17^A^
M2	20	348.13 ± 96 ^A^	376.89 ± 101^B^	5.89 ± 0.27 ^B^
PTN	20	387.75 ± 144^A^	338.68 ± 112^B^	5.77 ± 0.34 ^B^
SS File	20	317.45 ± 86 ^A^	355.98 ± 96^B^	5.83 ± 0.26 ^B^
P value*		0.171	< 0.001	< 0.001

*Different letters indicate statistically significant differences

## Discussion

In this study, slender curved roots were selected to represent the worst-case scenario clinically in determining the effect of root canal instrumentation on the structural integrity of fragile roots. Two contemporary rotary NiTi systems were selected to represent the two main root canal preparation philosophies of small apical size and large taper (PTN—25/0.06) versus larger apical size and conservative taper (M2—35/0.04). The hand instrumentation group preparations are based on traditional step-back techniques resulting in an overall taper of 25/0.05. The latter are more likely to vary dimensionally compared with machined NiTi preparations that are likely to be more standardised.

The statistically significantly highest FR (Table 1) was observed in the control group but there was no significant difference between the instrumentation groups. Several studies have examined the mean FL required to fracture mandibular first and second molars [14–16]. Lam et al. [14] observed no significant differences in the mean FL for K-files, Light-Speed (Light-speed Technology Inc., San Antonio, TX) and Greater Taper NiTi instruments (Tulsa Dental Products, Tulsa, OK), but FR was not calculated. Furthermore, Lertchirakarn et al. [15] and Lindauer et al. [16] reported mean FL for mesial roots of mandibular molars, but the former was a finite element analysis not based on canal instrumentation, and the latter assessed hand and ultrasonic preparations not involving standardised tapers. Another study [1] found that greater taper instruments removed more root dentine than those with hand instruments, and the former were more susceptible to fracture.

The conflicting results of the FR of instrumented roots could be attributed to factors related to the method of calculation. Where other studies have considered only the maximum load to fracture [9, 14–16], the present study determined FL but also accounted for the variability of the size of the roots and volume of the remaining dentine to mathematically calculate true FR. Previous studies [9, 11, 17, 18] standardised the specimens by selecting teeth with similar root length, radiographic dimensions (buccolingual and mesiodistal) or the weight of the roots. The weights of the roots had a medium correlation with fracture loading while multiplication of the buccolingual and mesiodistal dimensions had a low correlation with fracture loading [11]. Some authors have reported that the dimensions of the instrumented roots such as length and width, the angle at which the force is applied to the root, shape and size of the steel tip, mounting configuration of the root in the acrylic block and obturation method are all factors affecting the predisposition of vertical root fracture [8, 9, 19, 20].

In the present study, although roots selected had similar length and mesiodistal and buccolingual diameters, the canal shape and taper varied as different instrumentation methods were employed. Therefore, to account for confounding variables such as root length, volume and canal taper, these factors can be considered by calculating the FR (compressive strength) rather than reporting only the maximum FL. An important methodological aspect incorporated in the present study design was considering the root surface area and volume. The significant differences between the FL and FR data confirmed different values when root surface and volume are considered. While there were no differences in the FL, the FR of the roots varied with the volume and size. Applying the formula to the root canal preparation shape was a potential limitation in this study because the final canal preparation shape may well not be identical to the geometrically predicted shape. However, the pre-operative selection of teeth aimed to select those teeth with radiographically mature root canal systems which were confirmed once instrumentation was commenced. There will always be irregularities and non-instrumented regions within any root canal system [21], but this would apply to all the samples in this study, so the effect would have been minimized. Furthermore, the root curvatures did not match the geometric shape of a truncated cone but if the latter were bent to any degree the volume and surface area would remain the same and this also applied to all the teeth in this study.

Therefore, the results of the present study indicate that reporting only the maximum FL may be misleading because the root surface area and volume made a significant difference to the outcome. This difference can be explained by the fracture mechanics [22] because the strength of the roots is not just a function of the load applied, but also the size, shape and microstructure of the tooth. While the three forms of canal instrumentation in this study weakened the roots, there was no significant difference between the different canal instrumentation philosophies, although there seemed to be a trend toward smaller tapers.

## Limitations

A limitation of this study was that the method did not take into account the varying stress distribution within each sample, such that the usefulness of the technique may be limited to similar loading scenarios. However, this loading scenario is often used by researchers to simulate root fracture resistance and is considered standard. It should also be noted that the canal taper of the control group was estimated to be 0.02, but a non-instrumented canal may have irregularities, the effects of which were considered negligible in the present study. Also, exposing and immersing the specimens in chemicals (NaOCl and chloramine T) may adversely affect dentine structure, but this applied to all the teeth in the study.

## Conclusions

Within the limitations of this study, there seemed to be a direct and significant correlation between the root volume and canal taper with FR which was not identified if considering only FL. The trend of larger apical size and smaller taper resulting in greater FR needs to be confirmed with further research considering different root anatomy.

## Data Availability

The datasets used and/or analysed during the current study available from the corresponding author on reasonable request.

## Abbreviations

SS: Stainless steel hand files
NiTi: Nickel-titanium
PVC: Polyvinyl Chloride
N: Force to fracture
FL: Maximum load
FR: Fracture resistance
SPSS: Statistical Package for the Social Sciences
WL: Working length
NaOCl: Sodium hypochlorite
GG: Gates glidden
PTN: ProTaper Next
M2: Mtwo
GP: Gutta-percha
CEJ: Cementoenamel Junction
V1: Total volume of the root
V2: Volume of the root canal space
ANOVA: Analysis of variance
